# Which kind and how much physical activity?

**DOI:** 10.1186/1824-7288-40-S1-A23

**Published:** 2014-08-11

**Authors:** Ugo Giordano

**Affiliations:** 1 Ospedale Bambin Gesù, Roma, Italy

## 

Physical fitness is important for the optimal development both in children and adolescents. Fitness programs for youth should be recommended and encouraged.

Prior to participation in sport program a detailed medical examination should be performed including an ECG at rest.

It’s important that children should be exposed to a wide variety of sporting activities to discover which is the best for them to find out what they like most and to figure out which is best suited to their auxological condition. The rules and duration of physical activities should be appropriate to the age of the participant and excessively long distance (competitive) running events are not recommended for children prior to maturation.

Important is the difference between competitive sport and not competitive, both in terms of efforts as hours per week as involvement in physical stress.

Sports activities are necessary also to prevent chronic diseases like obesity, arterial hypertension, cardiovascular disease, hypercholesterolemia, diabetes and anxiety.

Good health or good physical condition is guaranteed by a healthy lifestyle rather than a genetic inheritance. Few but very important are the factors that contribute to a good aging, including physical movement.

